# Opinion and Sentiment Analysis of Palliative Care in the Era of COVID-19

**DOI:** 10.3390/healthcare11060855

**Published:** 2023-03-14

**Authors:** Megumi Inoue, Meng-Hao Li, Mahdi Hashemi, Yang Yu, Jahnavi Jonnalagadda, Rajendra Kulkarni, Matthew Kestenbaum, Denise Mohess, Naoru Koizumi

**Affiliations:** 1 Department of Social Work, George Mason University, 4400 University Drive, MS 1F8, Fairfax, VA 22030, USA; 2 Schar School of Policy and Government, George Mason University, Arlington, VA 22201, USA; 3 Department of Information Sciences and Technology, George Mason University, Fairfax, VA 22030, USA; 4 Capital Caring Health, Falls Church, VA 22042, USA; 5 Yale New Haven Health System, Bridgeport, CT 06610, USA

**Keywords:** palliative care, COVID-19, Tweets, information

## Abstract

During the COVID-19 pandemic, the value of palliative care has become more evident than ever. The current study quantitatively investigated the perceptions of palliative care emerging from the pandemic experience by analyzing a total of 26,494 English Tweets collected between 1 January 2020 and 1 January 2022. Such an investigation was considered invaluable in the era of more people sharing and seeking healthcare information on social media, as well as the emerging roles of palliative care. Using a web scraping method, we reviewed 6000 randomly selected Tweets and identified four themes in the extracted Tweets: (1) Negative Impact of the Pandemic on Palliative Care; (2) Positive Impact of the Pandemic on Palliative Care; (3) Recognized Benefits of Palliative Care; (4) Myth of Palliative Care. Although a large volume of Tweets focused on the negative impact of COVID-19 on palliative care as expected, we found almost the same volume of Tweets that were focused on the positive impact of COVID-19 on palliative care. We also found a smaller volume of Tweets associated with myths about palliative care. Using these manually classified Tweets, we trained machine learning (ML) algorithms to automatically classify the remaining tweets. The automatic classification of Tweets was found to be effective in classifying the negative impact of the COVID-19.

## 1. Key Message

The study collected Tweets from 1 January 2020–1 January 2022 that referred to palliative care in relation to the pandemic. We found Tweets that were focused on both the negative and positive impacts of COVID-19 on palliative care. We also found Tweets that were associated with myths about palliative care.

## 2. Introduction

Palliative care is specialized, holistic, person-centered medical care that aims to manage the symptoms of serious illnesses and to improve the quality of life of patients, their families, and their caregivers [1]. It provides communication, care coordination, and emotional support to patients and their family members, in addition to symptom management [2]. Palliative care seeks to align patients’ goals with the potential added benefit of lowering healthcare costs [3]. Palliative care use is associated with fewer ER visits, reduced undesired and unnecessary treatments, and decreased hospitalizations, which results in a healthcare cost reduction of USD 4258 for home-based care per person per month [3]. Early palliative care consultation is reported to achieve USD 1539 overall cost savings per hospitalization [4]. Over the past decade, there have been various public and private efforts to promote palliative care in patients and healthcare systems, including the establishment of palliative medicine as a specialty, community education, and funding opportunities for nonprofit service organizations and research institutions.

Despite the new movements, palliative care is still underutilized, and the timely identification of patients who would benefit from palliative care is an ongoing challenge [5,6]. One of the reasons for this underutilization is a lack of public recognition of palliative care and an understanding of its value [7,8]. Research using a nationally representative U.S. sample found that 70.2% of respondents had never heard of palliative care [8]. Among those who had heard about it, few had an accurate understanding of it [8]. Some examples of common misconceptions include the following: palliative care and hospice/end-of-life care are the same; receiving palliative care means giving up treatment; palliative care hastens death; palliative care is only available in the hospital; and palliative care leads to addiction to pain medicines [8,9,10]. Inaccurate knowledge is a barrier to promoting palliative care.

### Palliative Care during COVID-19 Pandemic

During the COVID-19 pandemic, the value of palliative care has become evident as a mechanism to facilitate treatment decisions in support of patients in various care facilities and their families [11]. Older adults with multi-morbidity have experienced the most severe forms of COVID-19 symptoms, including trouble breathing and death [12]. Palliative care could provide communication, care coordination, and emotional support to these patients and their family members, in addition to symptom management [2]. In order to further promote palliative care use, especially in the context of future pandemics, it is important to investigate the types of opinions and perceptions about palliative care that people exchanged in relation to the COVID-19 pandemic, including misconceptions. Prior research on the misconceptions largely relied on a survey-based approach. Since an increasing number of people share their health information and seek healthcare information on social media, where misinformation can quickly spread, it is critical to investigate the types of information about palliative care circulating on social media. The pandemic has amplified how quickly information and opinions spread on social media and how such information impacts people’s behavior [13,14].

The current study focused on Twitter, the most popular social media platform for healthcare communication [15]. A few faculty members with expertise in palliative care (hereinafter referred to as “domain experts”) and a review team reviewed and identified four themes in the extracted Tweets. Further, a Long Short-Term Memory (LSTM) Network, a novel machine learning (ML) algorithm, was applied to train four ML models that could potentially automate the Tweet classification in the future. Such models are expected to help policymakers to promptly analyze the public opinions on palliative care in response to public health emergencies.

## 3. Methods

### 3.1. Tweet Collection and Filtering Based on Time, Keywords, and Location

We searched original Tweets (not re-tweets, replies, or quotes) containing the word “palliative” and at the same time one of the following three words: “COVID”, “corona”, or “coronavirus”. The search for Tweets posted between 1 January 2020 (approximately marking the beginning of the Coronavirus pandemic) and 1 January 2022 resulted in 26,494 Tweets. The Twitter API was used to retrieve these Tweets. In more detail, a direct request was sent to the Twitter server using its API, asking for Tweets with the aforementioned temporal constraints that included the aforementioned keywords. The Twitter server would respond with a JSON file that contained the Tweets. In the JSON file, each Tweet was represented separately with pairs of attributes and values. Attributes were, for instance, the text, author, and time of the Tweet. To make it easier for our classifiers to manually classify these Tweets, we then automatically transformed this JSON file into an Excel sheet, where each row would represent a Tweet and its attributes. This Excel sheet was used to manage and process the data over the next steps. Two domain experts reviewed the first 200 Tweets to find that a significant number of the extracted Tweets did not contain any perceptions or opinions on palliative care. Thus, we thereafter divided Tweets into “related Tweets” and “unrelated Tweets”. “Related Tweets” contained information or opinions regarding palliative care, while “unrelated Tweets” included advertisements for palliative care seminars, scientific articles, and conferences, or palliative care doctors and nurses commenting or reporting on the COVID-19 situation. Then, the domain experts conducted a thematic analysis on the “related Tweets”, starting by familiarizing themselves with the content of Tweets by reading and re-reading them. After each person identified the initial codes independently, they discussed their codes until they reached an agreement. This process yielded three initial themes: recognized benefits of palliative care, the impact of the pandemic on palliative care, and myths.

Due to the limited number of students and staff who were available to review the Tweets, we randomly selected 6000 out of the 26,494 downloaded Tweets. The two domain experts, two research staff, and six student research assistants then manually reviewed the 6000 Tweets to solidify the themes and then classify them into the final themes. In the beginning, an hour-long information session was held by the domain experts to train other members of the research team on the Tweets’ classification. Then, the domain experts formed three smaller review teams, each of which was led by a senior member of the research team (i.e., either a domain expert or a post-doctoral researcher working under the domain experts) to ensure consistency and accuracy of classification. Each Tweet was independently reviewed by one senior and one to two other team members depending on their availability. Those who reviewed the same Tweet compared and discussed their classifications until they reached an agreement. The inter-rater reliability was 86.28%. When reviewers encountered uncertainty or disagreement, Tweets were brought to a full team meeting for discussion. Throughout the process, decision criteria were further polished to improve the accuracy. During the process, the review team recognized that a large proportion of Tweets were about the impact of the pandemic on palliative care. In addition, such impacts included both negative and positive impacts of the pandemic. Consequently, four final themes were identified: (1) *Recognized Benefits of Palliative Care*; (2) *Positive Impact of the Pandemic on Palliative Care*; (3) *Negative Impact of the Pandemic on Palliative Care*; and (4) *Myths of Palliative Care* (see Table 1).

**Figure 1:**
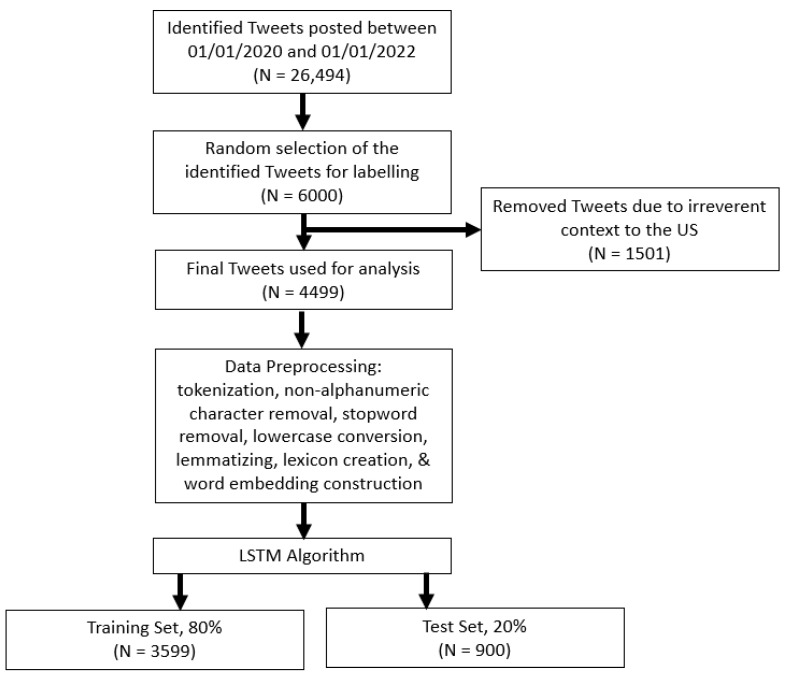
PRISMA diagram for Tweet sample extraction.

During the review process, the team also learned that most Tweets from outside the U.S. referred to their local situations, without any relevance to palliative care. For instance, many Tweets that had the phrase “COVID palliative” were used to criticize the Nigerian government’s lack of support in controlling the pandemic. This finding led to the decision to focus only on the U.S. Tweets for the current study. To filter out the Tweets of a non-U.S. origin, we applied the two-step approach. First, we looked at the location description in the author profile of each Tweet to automatically detect U.S. locations based on this description. A subsequent manual investigation demonstrated that this filtering approach achieved accuracy of 99%. Second, we focused on those Tweets whose authors were located in the U.S. Consequently, we were left with 4499 Tweets as a final set (see Figure 1).

### 3.2. Natural Language Processing to Create Word Embeddings, or Feature Vectors

The input to a machine or automatic classifier is a feature vector. In other words, machines classify feature vectors. Therefore, each word needs to be converted to a feature vector. Natural language processing techniques address this need. The following steps are taken, in the order mentioned, to construct a feature vector out of each term: tokenization [1], non-alphanumeric character removal [2], stop word removal [2,3], lowercase conversion [2], lemmatizing [4] (instead of stemming [5]), lexicon creation, and feature vector or word embedding construction.

Tokenization is the process of transforming a stream of characters into a stream of processing units called tokens, e.g., syllables, words, phrases, or sentences [1]. Our tokenizer splits each text into words, i.e., words are tokens. Stop word removal entails removing non-informative words such as propositions, conjunctions, and certain high-frequency words.

The goal of both stemming and lemmatization is to reduce inflectional forms and derivationally related forms of a word to a common base form. For instance, car, cars, car’s, and cars’ will become car after either stemming or lemmatizing. Stemming (e.g., Porter’s stemming algorithm [5]) is a crude heuristic process that removes the ends of words and removes derivational affixes. Lemmatization, however, uses a vocabulary and analyzes words morphologically, to remove inflectional endings only and to return the base or dictionary form of a word, which is known as the lemma. Stemming collapses derivationally related words, whereas lemmatization only collapses the different inflectional forms of a lemma. Although stemmers use language-specific rules, they require less knowledge than a lemmatizer. A lemmatizer requires a complete vocabulary and morphological analysis to accurately identify the lemma for each word, which produces modest benefits for retrieval. Lemmatization is chosen over stemming in this work.

A lexicon is the vocabulary of a person, language, or branch of knowledge. In our study, the lexicon is the bag of unique lemmas that appear among the training samples/Tweets. The lexicon used for a classification task does not necessarily need to be a comprehensive vocabulary in that field. It only needs to be as comprehensive as the vocabulary of the training samples or corpus. The reason is that if a term does not appear in any of the training samples, including it in the lexicon will make no contribution in training the classifier, because the value of the term’s corresponding feature will be zero for all training samples. The size of the lexicon in our dataset was 15,728.

Finally, an embedding or feature vector of length 256 was assigned to each word in the lexicon, using the embedding layer offered by the Keras library in Python. The embedding vectors for each word were constructed using an iterative approach that attempted to assimilate the distribution of these vectors in the feature space to their distribution in the corpus. In other words, words that appeared close to each other in the Tweets would end up being close to each other in the feature space.

### 3.3. LSTM Classification

In ML, independent variables (i.e., predictors, explanatory variables, or input variables) are known as “features”, while dependent variables are known as “output variables”. In the current study, the output variables are the four identified themes, while features correspond to words in each Tweet. Each of the four output variables takes the value 0 (i.e., “negative class”) if the Tweet does not belong to the theme and the value 1 (i.e., “positive class”) otherwise. The current study applied a LSTM network [16], a state-of-the-art architecture of recurrent neural network (RNN), to assign Tweets to the identified themes [1,2,3]. To this end, we split the final set of 4499 Tweets to two sets, i.e., 80% (3599) into the training and 20% (900) into the test sets. This proportion of the training vs. test sets approximately reflects the class distribution of the output variables. The details of algorithm specifications are described in Appendix A. Recall, precision, and F1 score were used as the measures for the classifier’s accuracy [17]. Recall is defined as the number of true positive tweets divided by the total number of actual positive tweets. Precision is defined as the number of true positive tweets divided by the total number of predicted positive tweets. F1 score is the harmonic mean of recall and precision (i.e., 2 × Precision × Recall/(Precision + Recall)) [17]. A high F1 score indicates high precision and recall.

## 4. Results

### 4.1. Theme Distributions of Manually Classified Data

The distribution of the 4499 Tweets manually classified into the four themes is summarized in Table 2. In the data, the majority of Tweets were “unrelated” (80.7%) to any of the themes. Tweets classified as *Negative Impact of the Pandemic on Palliative Care*, *Positive Impact of the Pandemic on Palliative Care*, and *Recognized Benefits of Palliative Care* shared 7.8% (N = 350), 5.6% (N = 254), and 7.3% (N = 329), respectively. Tweets that fell under the theme of *Myths of Palliative Care* shared only 0.5% (N = 21) (Table 2). These themes were not mutually exclusive, i.e., some of the Tweets were classified into more than one theme.

### 4.2. Word Importance and Representative Tweets

The following subsections provide some representative Tweets seen under each theme.

#### 4.2.1. Negative Impact of the Pandemic on Palliative Care

Most Tweets in this theme were about the shortage in palliative care, delayed services/treatment for palliative care patients, and changes in hospital safety rules that have negatively affected palliative care patients and their families. Examples of such Tweets are listed below.

(1) doctors and nurses are dealing hostility and threats from patients upset over hospital safety rules due to the #COVID19 pandemic. @kenceemd, hospitalist & palliative care physician, discusses her experience at #uofuhealth
(2) such an emotionally draining week on placement, so many palliative cancer patients who were diagnosed late or had their surgery/chemo dates pushed back due to COVID
(3) shortage of palliative care in usa could amplify suffering for coronavirus patients https://t.co/5nzcggqzie (accessed on 29 January 2023) via @usatoday

#### 4.2.2. Positive Impact of the Pandemic on Palliative Care

Most Tweets in this theme were about the adoption of new technology such as telemedicine being available to palliative care patients, home-based care agencies’ incorporation of palliative care, and the development of palliative care toolkits and guidelines for nonpalliative care clinicians and medical staff. An expansion of palliative care services and renewed demand for such expansion were also viewed as positive for the field of palliative care and its patients. Examples of such Tweets are listed below.

(1) long-haul #COVID renews push to expand #palliativecare
(2) #telemedicine—it is saved so many chronically ill people from risking COVID to see their drs. it is also saves time & money—for me, it is often a 3 h one way drive to see my palliative care doctor. it is #somethingpositiveabout2020 that i hope will continue long after COVID
(3) coronavirus may push more home health providers into palliative care

#### 4.2.3. Recognized Benefits of Palliative Care

Most Tweets in this theme were about the acknowledgement of the benefits of palliative care. Many Tweets mentioned the importance of palliative care and how it is helpful in addressing severe symptoms and challenges brought by the pandemic. Examples of such Tweets are listed below.

(1) palliative care meets emotional needs during COVID-19
(2) the demand for palliative care has grown substantially during the pandemic. our director of palliative care rhonda gaugh, do explains what palliative care is and how it helps COVID patients.
(3) palliative care is an essential service to relieve pain and suffering related to COVID-19 and pre-existing health conditions
(4) palliative care puts compassion into the COVID crisis, it adds the humanization, it put the patient in the center of the crisis and not other issues

#### 4.2.4. Myths of Palliative Care

Tweets found in this theme were predominantly about the anger towards people with anti-vaccination attitudes who occupy hospital beds. Tweets indicated that these people should only be given “palliative care” instead of being treated, implying that receiving palliative care means giving up treatment, which is one of the most well-recognized misconceptions about palliative care [8,9]. There were Tweets that expressed disagreement with counting a palliative care patient’s death as a COVID-19 death, mentioning that the patient was already receiving palliative care. This also implies that patients receiving palliative care are dying, which is another well-recognized misconception [8,9]. Some examples are as follows:

(1) i wish hospitals would refuse to treat non vaccinated COVID patient, palliative care only or ivermectin/hydroxychloroquine if they like. why are we bothering with people who have chosen to become infected and suffer adverse results?
(2) at this point when you ask a newly admitted patient if they have been vaxxed for COVID and they say “no”, just offer them palliative care and send them home.
(3) “theoretically, a 90-year-old cancer patient already on palliative care could die but have coronavirus in their system at the time of death that could be recorded as a coronavirus death.”

#### 4.2.5. Unrelated

The majority of unrelated Tweets involved palliative care related to advertisement. The following list outlines the types of unrelated Tweets.

(1) Advertisement of webinars, YouTube videos, or journal articles, e.g., “today’s palliative care discussion series features guest xxx” or “visit the capc youtube channel for the latest videos on palliative care messaging…”
(2) Palliative care was mentioned but not as its main point or focus, i.e., “over the past week my mother almost died from COVID. she was unvaccinated. she was hospitalized and didn’t want to be intubated, but managed to pull through and her oxygen levels rose, she’d planned to go palliative care. now she’ll live. please get the vaccine” or “trump touted hydroxychloroquine as a preventative treatment. the report states it was only helpful in severe cases when patients were on ventilators. so not preventive, palliative!”
(3) Opinions or perceptions that are not uniquely associated with palliative care, i.e., opinions that are commonly shared in the settings other than palliative care, including “i work in a long term facility in the dementia and palliative care unit. we are COVID free but in full ppe gear every day because co-workers refused to get vaccinated. i am so disgusted”

### 4.3. Model Performance of LSTM

Due to the small number of Tweets classified under the theme of *Myths of Palliative Care* (N = 21; 0.5%), LSTM could not be applied to this theme. The LSTM classifications were performed for the remining three themes. The proportion of the positive class (i.e., class 1) Tweets was small, ranging between 0.5% and 7.8%, for all themes. This highly skewed proportion towards the negative class (i.e., class 0) often leads to the overclassification of the Tweets into the negative class by the model, resulting in erroneously high prediction accuracy stemming predominantly from the correct classification of the negative/unrelated Tweets. To take this bias into account in the performance assessment, we used the recall, precision, and F1 score as the measures of the model’s performance [17].

Table 3 summarizes the performance metrics for the three themes. The highest recall, precision, and F1 score were achieved in predicting *Negative Impacts of the Pandemic*, followed by *Positive Impacts of the Pandemic*, *Recognized Benefits of Palliative Care*, and *Myths of Palliative Care*. Specifically, recall of 97% for *Negative Impacts of the Pandemic* indicates that the model mislabeled only 3% of Tweets under this theme as the negative class. Precision of 95% for *Negative Impacts of the Pandemic* means that only in 5% of cases, the model incorrectly classified a Tweet as the positive class, i.e., as related to the theme. This is considered to reflect the great performance of the model for such an unbalanced dataset. For *Positive Impacts of the Pandemic*, the recall score was reduced to 41%, while the precision score remained relatively high at 68%. For *Recognized Benefits of Palliative Care*, the recall falls to 27% and the precision to 55%, meaning that the model is ineffective in predicting both classes of Tweets.

## 5. Discussion

This study explored the types of perceptions and opinions on palliative care shared on Twitter in the COVID-19 era. Using a conventional web scraping method, we collected relevant Tweets and identified four themes in the extracted Tweets, i.e., *Negative Impact of the Pandemic*, *Positive Impact of the Pandemic*, *Recognized Benefits of Palliative Care*, and *Myths of Palliative Care*.

Surges in COVID-19 patients increased the demand for palliative care [18]. This increased demand created challenges in ensuring quality care for palliative care patients and providing timely services/treatment to them. Visitor restrictions adopted by medical facilities often prohibited family members from seeing their loved ones receiving palliative care. Tweets in the theme of *Negative Impacts* reflected these difficulties brought by the pandemic. While there were many Tweets pointing out the detrimental effects of the pandemic, our analysis also identified some positive impacts of the pandemic. In order to extend palliative care expertise to meet the surging demand and address challenges brought by the pandemic, various efforts were made and new strategies were put in place. One of the positive elements emerging from the pandemic experience is the increased implementation of telemedicine. Telemedicine has enabled patients to access palliative care while maintaining social distancing [19]. The development of toolkits and guidelines for palliative care is another positive impact captured by our analysis. These strategies developed under the pandemic have been distributed to nonpalliative care clinicians in healthcare agencies, which could expand the access to palliative care in various settings [20].

Our study also revealed that the pandemic helped to verify the value of palliative care. A number of Tweets posted by family members and healthcare workers acknowledged how palliative care had been useful and helpful for people suffering from COVID-19 infection. Such positive views on palliative care shared via Twitter must have had some impact on others who did not know much about palliative care. The Tweets related to misconception and/or misinformation were mainly associated with two well-reported misconceptions: that palliative care and hospice/end-of-life care are the same, and that receiving palliative care means giving up treatment [8,9]. Although such Tweets were fewer in number, they could still have an impact by being re-tweeted and further circulated. The following Tweet, for instance, signifies the negative impact of such Tweets:

“no one is trying to ‘cure’ me. i’m afraid to go to the hospital if i get sick or hurt right now because, my chart has been labeled, by the drs, as ‘palliative care’. not usually an issue, but now may be. the COVID death numbers are wrong, not counting all”.

This person is afraid of going to hospital and being denied treatment when becoming sick due to being a palliative care patient. This is just one example of the unfortunate implications of misinformation. As Twitter currently does not screen content, misinformation can spread quickly and widely.

This study has several limitations. First, our analysis only included original Tweets, so the way in which these original Tweets were further circulated is unknown. Future research needs to examine re-tweets, particularly for those Tweets that included myths, which will offer a more comprehensive picture of the information shared. Second, Tweet data are inherently susceptible to sample selection bias. Nevertheless, the results presented in the current study need to be interpreted with a cautionary note as they are more likely to represent the views and perceptions of younger and technologically oriented individuals. Third, location information regarding where Tweets originated was largely unavailable. Previous research has reported higher access to palliative care in the states that are racially homogeneous, higher in socioeconomic status, and more liberal [21]. Examination of regional variations in the distribution of the themes could offer an insight into the public’s perspective on palliative care. Fourth, it was challenging to identify and remove duplicated Tweets effectively unless Tweets were identical. Thus, Tweets with a similar context and authored by the same person could have been counted more than once, affecting the statistics. Fifth, there was a large volume of Tweets coming from a handful of foreign countries, such as Nigeria, Australia, the U.K., and Canada, which were excluded from the analysis. If we were to investigate the country variations, the collection of Tweets in local languages is warranted.

Finally, the current paper demonstrated some success in the automatic classification of Tweets using ML. Specifically, the LSTM Network-based algorithm was able to classify *Negative Impacts of the Pandemic* with high accuracy, suggesting that our future analysis could accommodate a much higher volume of Tweets to examine the time trend as well as the geographic variation of the *Negative Impact* Tweets. For instance, researchers could test whether the proportion of the *Negative Impact* Tweets is correlated with the palliative care resource availability or the policy to control the pandemic at the state/region/country level. Similarly, given that the majority of the *Negative Impact* Tweets were about the lack of access to the appropriate palliative care, as well as overwhelmed medical staff, the proportion of the *Negative Impact* Tweets could be used as a proxy measure to assess palliative care needs. For other themes, further training of the algorithm is warranted with more Tweets of these themes. The eventual goal of our endeavor is to investigate both time and geographic trends of different perceptions and opinions related to Tweets. With more Tweets related to palliative care, our algorithm could also be trained to identify subthemes under each theme or to detect latent themes that humans have failed to identify. Therefore, the use of Twitter analysis can be a valuable tool to inform public health policymaking in a variety of ways. Through monitoring public sentiments on Twitter, policymakers can gain insights into attitudes and opinions surrounding specific pandemic issues, such as vaccination, palliative care, or shelter-in-place, and use this information to guide policies and interventions. Analyzing tweets pertaining to social distancing behaviors, such as physical activity and masking, also offers policymakers the opportunity to identify trends and patterns, which can in turn help to shape more effective interventions. Moreover, Twitter’s ability to identify outbreaks of infectious diseases by tracking tweets that mention symptoms such as fever, cough, or sore throat has proved instrumental in helping policymakers to take prompt action and prevent the further spread of disease. Lastly, Twitter can help policymakers to identify vulnerable populations during the pandemic, such as those with limited access to healthcare or those at higher risk for certain health conditions, by analyzing tweets related to these populations. Armed with this information, policymakers can design targeted interventions and allocate resources more effectively, thereby improving public health outcomes.

## 6. Conclusions

The current study used web scraping and a machine learning algorithm to investigate perceptions and opinions related to palliative care in the era of COVID-19. Our study confirmed the prior literature stating that the value of palliative care became more apparent to the general public through the pandemic. Although a large volume of Tweets referred to the *Negative Impacts of COVID-19 on Palliative Care*, almost an equal proportion of Tweets were about the *Positive Impacts of COVID-19 on Palliative Care*. The study demonstrated that these positive impacts were primarily related to the emergence of telehealth use and toolkits, as well as the development of coordination protocols between palliative care providers and other medical service providers.

## Figures and Tables

**Table 1:** Definition of labeled topics.

Theme	Definition
Recognized Benefits of Palliative Care	Tweets acknowledge the importance or value of palliative care.
Positive Impact of the Pandemic on Palliative Care	Tweets state that something good happened or resulted from palliative care because of the pandemic, such as increasing recognition of palliative care in public and the development of toolkits to care for patients.
Negative Impact of the Pandemic on Palliative Care	Tweets voice concerns and difficulties in caring for palliative care patients, such as having overwhelmed medical staff, keeping patients isolated from their family members, and facing challenges to provide appropriate and timely treatment for palliative care patients.
Myths of Palliative Care	Tweets include misinformation and misconceptions, such as implying that palliative care means not providing appropriate treatment.

**Table 2:** Distribution of Tweets across themes.

Theme	N	%
Negative Impact of the Pandemic on Palliative Care	350	7.8
Positive Impact of the Pandemic on Palliative Care	254	5.6
Recognized Benefits of Palliative Care	329	7.3
Myths of Palliative Care	21	0.5
Unrelated Tweets	3631	80.7

**Table 3:** Model performance for the four outcome variables.

Outcome Variables/Theme	# of Test Samples	Size of the Positive (Class 1)	Size of the Negative (Class 0)	Recall	Precision	F1 Score
Negative Impact of the Pandemic	900	70	830	0.97	0.95	0.96
Positive Impact of the Pandemic	900	51	849	0.41	0.68	0.51
Recognized Benefits of Palliative Care	900	66	834	0.27	0.55	0.36

## Data Availability

Not applicable.

## Appendix A

Our LSTM network was composed of the following:

- One input layer, which receives the vectorized words of the Tweet.
- One hidden layer, also referred to as a memory cell, with 256 nodes and a dropout rate of 0.3, with a rectified linear unit (ReLU) activation function. The 0.3 dropout rate means that 30% of nodes in this layer will be ignored during each training epoch. This improves the network’s stability by increasing resistance to noise in the data. This layer produces a hidden state at every time step.
- One dense layer with 256 nodes and a dropout rate of 0.3. This layer receives the hidden state generated by the memory cell and produces a class label.
- One output layer with a SoftMax activation function, which produces a probability distribution over the classes. The SoftMax function, *softmax*(*z_i_*) = *exp*(*z_i_*)/*Ʃ_j_ exp*(*z_j_*), used in the final layer, transforms the values (*z_i_*) to normalized exponential probabilities whose summation is one (i.e., *Ʃ_i_ p_i_* = 1). There are as many units in the output layer as there are classes. Each class is locally represented by a binary target vector with one non-zero component.
- The cross-entropy cost function, applied in the network, is calculated as—*Ʃ_c_ y_c_ log*(*p_c_*), where *c* represents a neuron (or class) in the output layer, *y_c_* represents the desired value (0 or 1) at that class, and *p_c_* is the predicted probability at that class. The network is trained using the Adam optimization algorithm [22], which is an extension to the stochastic gradient descent (SGD) approach, with a batch size of 256. In other words, at each training epoch, only 256 training samples were used. Unlike SGD, which maintains a single and fixed learning rate for all synaptic weight updates, Adam continually adjusts individual adaptive learning rates for each synaptic weight based on estimates of the first and second moments of the gradients. A five-epoch rule is applied to stop the training, i.e., the training is stopped if the validation accuracy does not improve for five consecutive epochs and the most accurate version of the model is stored. The validation accuracy is the percentage of validation Tweets (i.e., Tweets that have not been used for training) that are correctly classified.
